# Amplified EQCM-D detection of extracellular vesicles using 2D gold nanostructured arrays fabricated by block copolymer self-assembly†

**DOI:** 10.1039/d2nh00424k

**Published:** 2023-01-24

**Authors:** Jugal Suthar, Alberto Alvarez-Fernandez, Esther Osarfo-Mensah, Stefano Angioletti-Uberti, Gareth R. Williams, Stefan Guldin

**Affiliations:** a Department of Chemical Engineering, University College London, Torrington Place London WC1E 7JE UK alberto.fernandez@ucl.ac.uk s.guldin@ucl.ac.uk; b UCL School of Pharmacy, University College London 29-39 Brunswick Square, Bloomsbury London WC1N 1AX UK; c Department of Materials, Imperial College London Exhibition Road London SW7 2AZ UK

## Abstract

Extracellular vesicles (EVs) are routinely released from nearly all cell types as transport vehicles and for cell communication. Crucially, they contain biomolecular content for the identification of health and disease states that can be detected from readily accessible physiological fluids, including urine, plasma, or saliva. Despite their clinical utility within noninvasive diagnostic platforms such as liquid biopsies, the currently available portfolio of analytical approaches are challenged by EV heterogeneity in size and composition, as well as the complexity of native biofluids. Quartz crystal microbalance with dissipation monitoring (QCM-D) has recently emerged as a powerful alternative for the phenotypic detection of EVs, offering multiple modes of analyte discrimination by frequency and dissipation. While providing rich data for sensor development, further progress is required to reduce detection limits and fully exploit the technique's potential within biosensing. Herein, we investigate the impact of nanostructuring the sensor electrode surface for enhancing its detection capabilities. We employ self-assembly of the block copolymer polystyrene-*block*-poly(4-vinylpyridine) to create well defined 2D gold islands *via* selective impregnation of the pyridine domain with gold precursors and subsequent removal of the template. When matched to the EV length scale, we find a 4-fold improvement in sensitivity despite a 4-fold reduction in area for analyte and ligand anchoring in comparison to a flat sensor surface. Creation of tailored and confined sensing regions interspersed by non-binding silica provides optimal spatial orientation for EV capture with reduced steric effects and negative cooperativity of grafted antibodies, offering a promising route for facilitated binding and enhanced performance of sensor platforms.

## Abstract 2

In this work, we are investigating the impact of tailoring the active sites of a biosensor surface to mirror lateral analyte feature sizes, here extracellular vesicles. We relate enhanced sensitivity to reduced steric effects and provide guidelines for future design.

## Graphical abstract

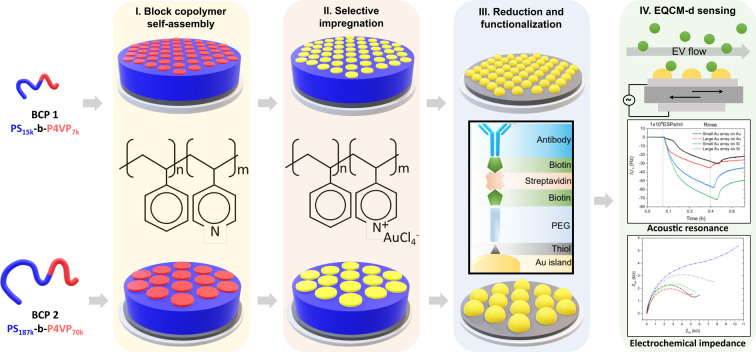

**New concepts**

Extracellular vesicles (EV) have become a promising source of biomarkers for disease diagnostics *via* liquid biopsies from readily accessible bodily fluids but challenges remain on the sensitivity, specificity and clinical implementation of current analytical workflows. In this work, we are investigating the impact of tailoring the active sites of the biosensor surface, herein the electrode of a quartz crystal microbalance with dissipation monitoring (QCM-D), to mirror lateral analyte feature sizes. We relate the enhanced sensitivity to improved surface anchoring due to reduced steric effects as a result of sensor nanostructuration and provide guidelines for design considerations.

## 1 Introduction

Extracellular vesicles (EVs) are nanosized circulating assemblies released from cells as transport vehicles and for cell communication.^1^ Crucially, they contain biomolecular content (proteins, nucleic acids, and lipids) that can provide an indication of health and disease status.^2,3^ This, combined with their ubiquity in accessible physiological fluids, including urine, plasma, or saliva, creates promising pathways to incorporate EVs in routine diagnostics, *e.g.* for various cancer types, acute brain injury, kidney or neurodegenerative diseases.^4,5^ Several analytical principles based on surface plasmon resonance (SPR), fluorescence, absorbance or electrochemical spectroscopy have been successfully implemented in the detection and characterization of EVs at clinically relevant concentrations.^6^ However, despite promising results, current approaches face a number of intrinsic challenges to EV characterisation, namely their heterogeneity in size and composition. As a result, the aforementioned strategies either require elaborate sample labelling complex experimental set-ups or lack specificity in being able to reliably discern between various EV subpopulations and artefacts, preventing their full clinical exploitation.

Analytical approaches based on quartz crystal microbalance with dissipation monitoring (QCM-D) have recently emerged as powerful alternative techniques for the phenotypic characterisation of EVs above their endogenous concentration, offering multiple modes of analyte discrimination (frequency and dissipation).^7^ Moreover, the sensitivity of this platform was further improved to 6.71 × 10^7^ EV-sized particles (ESP) per mL in complex media, *via* the addition of tandem electrochemical impedance spectroscopy (EIS) based detection of CD63-positive extracellular vesicles, as part of a dual modal electrochemical QCM-D analytical approach (EQCM-D).^8^ However, these reported detection limits are still considerably higher than competing analytical methods.

Strategic efforts to improve biosensing performance include the structural modification of the sensor surface at the nanoscale. A common underpinning rationale is to increase the detection surface area (surface-to-volume ratio) for enlarged binding capacity, or modify the surface aspect ratio (length-to-diameter ratio) for optimal ligand arrangement, collectively enhancing analytical sensitivity and specificity.^9–11^ Until now, nanostructuration of QCM-D sensor surfaces has been focused on creating 3D porous structures such as anodic aluminium oxide or inverse opals, showing promising results in improving analytical response against different targets, such as enzymes, liposomes, and antibodies.^12–15^ However, those nanofabrication methodologies are limited in providing access to tailored, reproducible, and precisely controlled structures, preventing full exploitation for clinical applications. Moreover, the use of porous thin films can lead to solvent and artefact entrapment effects in the nanostructured film, hindering data interpretation and introducing uncertainty over obtained results. In principle, surface structuration with out-of-plane features may be more desirable in the context of immunosensing, due to their propensity to support growth of orthogonal structures that are more accessible to analytes.^16,17^

Top-down lithographic techniques such as photo-lithography, electron beam lithography or nanoimprinting lithography have been successfully used to produce nanostructured surfaces for enhanced detection of EV proteins.^18–20^ Cai *et al.* reported a gold nano-checkerboard surface created using an interference lithography approach that displayed increased sensing performance.^21^ Similarly, Raghu *et al.* employed electron beam lithography to create elliptically-shaped gold nanopillars that delivered improved sensitivities by over three orders of magnitude.^22^ Bottom-up approaches to create suitable nanostructured surfaces include the random distribution of pre-synthesized colloidal nano-objects across the sensor surface. This method has been widely employed in electrochemical-based immuno-detection.^23^ Letchumanan *et al.* recently described the addition of gold-nanorods to enhance differential pulse voltammetry (DPV) detection of C-reactive protein by 100-fold.^24^ More complex architectures based on bimetallic nanopillar sensors have elicited a 10-fold increase in square wave voltammetry sensitivity towards insulin compared to commercial detectors, as a result of improved electron transfer across a larger working electrode surface area.^25^ Lithography free nano-patterning has also been used to increase the binding surface area of a device for higher ligand probe density and improved efficiency for EV CD24 detection.^26^ Nevertheless, limitations remain for top-down and bottom-up approaches, with the former requiring sophisticated and costly instrumentation and the latter challenged by reproducibility issues.

In the search for suitable synthetic strategies, block copolymer (BCP) self-assembly represents an attractive approach for the fabrication of a variety of tailored nanoarchitectures with high order and tunable periodicities over large surface areas, all in a reproducible, fast and cost-effective manner.^27,28^ Tuning of the block ratio enables control over the microphase-separated morphology, whilst tailoring the molecular weight offers precise adaptation of feature sizes across a lengthscale of 5 to 100 nm.^29^ The BCP templates can subsequently serve as etch masks or sacrificial templates to create nanometric metal and dielectric material patterns,^30–33^ using deposition strategies such as sputtering,^34^ electrochemical deposition,^35,36^ or sequential infiltration synthesis and aqueous metal reduction (AMR).^37–40^ AMR introduces metal (gold) in an aqueous environment and is reliant on the chemical interaction between metal ions and a particular BCP phase of the template.^41,42^ After incubation and successful impregnation, the BCP scaffold may then be removed through reactive ion etching (RIE), UV/Ozone exposure or chemical agents.^43^

While the principle of selective hybridization of self-assembled BCP nanostructures provides pathways to bespoke surface engineering of metallic patterns, this method has not been extensively investigated for biosensor design, especially in the context of QCM-D. Herein, two poly(styrene)-*block*-poly(4-vinylpyridine) (PS-*b*-P4VP) BCPs with bespoke molecular weights are employed in the fabrication of well-ordered and size-tailored Au nano islands across the sensor surface. Au arrays are deployed on both silica and gold coated sensors, to explore the impact of surface composition, confinement and arrangement on the analytical performance towards CD63-positive EVs using previously established QCM-D and EQCM-D techniques. Complementary surface characterisation of sensor substrates is conducted to investigate the impact of Au NP-array periodicity, size on the binding surface area and spatial orientation, thus elucidating the underlying mechanisms behind the changes witnessed in analytical performance.

## 2 Results and discussion

### 2.1 BCP self-assembly and Au NP formation

**Fig. 1:**
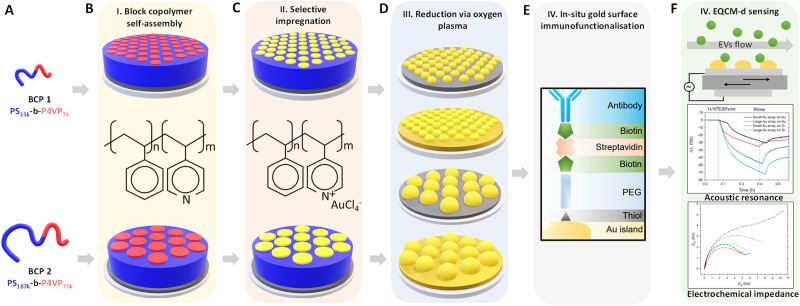
Schematic summary of the experimental approach. (A) BCPs used during this work. (B) BCP self-assembly on the piezoelectric sensors. (C) Selective impregnation with metallic Au precursors. (D) Removal of the BCP template and Au reduction by O2 plasma. (E) Immunofunctionalization of the nanostructured surfaces with anti-CD63 antibodies for specific surface capture of extracellular vesicles. (F) Evaluation of QCM-D and EQCM-D performance of the created substrates in the detection of CD63-positive extracellular vesicles.

Au NP arrays with controllable size and interdistances were fabricated following the methodology illustrated in Fig. 1. As a first step, two different molecular weight (*M*_w_) BCPs were deposited and self assembled on top of the QCM-D sensor by spin-coating. Subsequently, metallic Au NPs were created by selectively impregnating the BCP thin films with metallic gold precursors, followed by their reduction to metallic gold and the removal of the BCP scaffold *via* O_2_ plasma treatment. In a final step, the analytical performance of the nanostructured sensors towards CD63-positive EVs was evaluated by QCM-D and EQCM-D.

**Fig. 2:**
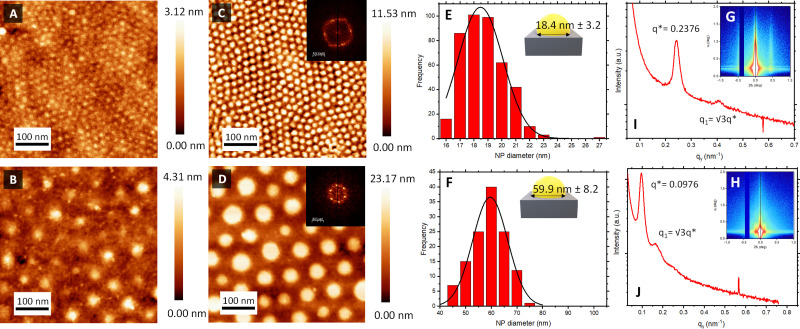
Structural characterisation of sensor surfaces. AFM micrographs following casting of (A) BCP1 and (B) BCP2 illustrating self-assembled out-of-plane cylinders. AFM micrographs of formed Au NP islands following impregnation and template etching of (C) BCP1 and (D) BCP2. Scale bars: 100 nm. FFT of both images are shown in the respective inset. Corresponding particle diameter size distribution of (E) BCP1 and (F) BCP2 nanoarrays *via* image analysis of AFM micrographs. GISAXS profile of a silicon wafer with (G) BCP1 Au NPs and (H) BCP2 Au NPs after O_2_ RIE etching. Out-of-plane scattering profiles corresponding to a horizontal line cut as a function of *q*_*y*_ for (I) BCP1 Au NP and (J) BCP2 Au NP arrays.

Fig. 2A and B shows the AFM topographical images obtained immediately after BCP deposition onto bare silica and gold QCM sensors. In both cases, out-of-plane poly(4-vinyl pyridine) (P4VP) cylinders in a poly(styrene) (PS) matrix were obtained directly after spin coating. The structure formation is promoted by the use of a solvent that was neutral for both blocks (propylene glycol methyl ether acetate, PGMEA). Furthermore, its high boiling point optimised the diffusion of the polymer blocks during the spin-coating process, leading to well-defined and ordered structures.^44^ Measurements after spin-coating showed a film thickness (*t*) of 35 nm for BCP1 and 75 nm for BCP2 respectively. As expected, the control over the macromolecular characteristics of the BCPs supported the tunable fabrication of nanostructures downstream. Thus, while BCP1 led to a hexagonal cylindrical assembly with a centre-to-centre distance (*D*_c–c_) of 21 nm, the higher *M*_w_ of BCP2 exhibited enlarged *D*_c–c_ of 76 nm (Fig. S1, ESI†).

In a subsequent step, obtained BCP films were used as a template for the fabrication of Au NP arrays. This process started with the selective impregnation of the P4VP cylinders with gold metallic precursors *via* AMR. This fabrication strategy is based on the selective ionic interactions between one of the polymer blocks and the metallic ions present in an aqueous solution.^39,40^ In our case, the Brønsted base character of the P4VP units allowed their selective hybridization with [AuCl_4_]^−^ ions, *via* immersion of the films in a HAuCl_4_ aqueous solution.^45^ After impregnation, BCP scaffolding was removed with O_2_ plasma, which led to the concurrent reduction of the Au^III^ ions to metallic gold.^40,46^

Atomic force microscopy (AFM) topographical images in Fig. 2C and D confirmed the successful formation of the Au NPs arrays on top of the piezoelectric sensors, reproducing the initial out-of-plane cylindrical BCP assembly arrangement. The reduction of the HAuCl_4_ salt into metallic dots was consistent with spectroscopic ellipsometry measurements (Fig. S2, ESI†), where the evolution of the measured *Ψ* and *Δ* as a function of the photon energy revealed a clear S-shaped feature in the region ≈2.4 eV, corresponding to the characteristic localised surface plasmon resonance of the metallic Au NP array. Power spectral density (PSD) from the captured AFM images revealed the consistency and stability of the created nanostructures across the fabrication process (Fig. S1, ESI†). Thus, interdomain spacing of the created Au NPs using BCP1 and BCP2 remained constant at 21 and 78 nm, respectively, across the extended regions of the sensor surface.

Another important parameter to consider is the diameter of the created Au NPs. Feature sizes were estimated by image analysis of the AFM micrographs (Fig. 2). Au NPs produced from the BCP1 template (BCP1 NPs) were estimated to have a modal diameter of 18.4 nm (± 3.2 nm) (Fig. 2E), whereas the ones obtained using the higher *M*_w_ BCP2 as a template (BCP2 NPs) exhibited a modal diameter of 59.9 nm (± 8.2 nm) (Fig. 2F). Combining these lateral dimensions with the NP heights obtained from the AFM topographical profiles (Fig. S3, ESI†) gave an estimated height-to-width ratio of 0.38 and 0.33 for BCP1 NPs and BCP2 NPs respectively, in line with a semi-ellipsoidal NP shape.

Grazing incidence X-ray scattering (GISAXS) experiments confirmed the structural order of the Au NPs arrays obtained from BCP1 and BCP2 templates (Fig. 2G and H respectively). In both instances, the patterns displayed intense Bragg peaks along *α*_*f*_ that are consistent with the presence of Au NPs on a silicon wafer surface. The horizontal line cut (out-of-plane) of the *q*_*y*_ component, integrated around the Yoneda band, represent the GISAXS intensity distribution parallel to the surface and are presented in Fig. 2I and J.^47^

The pronounced modulation of scattering intensity in this region is correlated to the interference effects induced by the surface roughness of the substrate. The *D*_c–c_ between the Au NPs can be calculated with respect to the position of the first Bragg peak (q*).^48^ Thus, for the smaller BCP1 NPs, the q* position of 0.247 nm^−1^ for the first Bragg peak indicates a *d*_c–c_ of 27.1 nm between NPs. Higher order Bragg peaks were observed at 
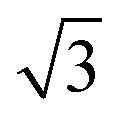
 consistent with an hexagonal ordering of the Au NP structure. Similar results were obtained for the larger BCP2 NPs. In this case, the position q* = 0.097 nm^−1^ of the first Bragg peak confers a *d*_c–c_ of 74.3 nm. These results aligned strongly with the PSD analysis presented in Fig. S1 (ESI†).

With the inter NP and planar dimensional information established, an estimation of the available binding surface area was made. As presented in Fig. S4A and B (ESI†), the radii and particle height acquired from Fig. 2 and Fig. S3 (ESI†) were used to approximate the surface area of a single Au NP (determined as half an oblate spheroid). Combining this with AFM-derived particle density estimations, the binding surface area across the sensor as a whole was evaluated. BCP1 Au NPs displayed nearly 7-fold higher density compared to the BCP2 Au NPs (398 NPs *vs.* 57 NPs per 500 nm^2^). Thus, BCP1 NP arrays offer a 2.7 higher surface binding area than BCP2 NP arrays. In return, the higher density of BCP1 NPs arrays reduced the contribution of the underlying flat substrate (46.2% of total surface area) compared to BCP2 Au NPs (76.8%). An important consideration of the herein presented approach is the material of the underlying flat substrate. For silica substrates, the Au NPs become effective confined binding arrays interdispersed by non-binding inter-particle regions. A gold substrate could act as a continuous binding surface in tandem with Au NPs and thus potentially compete for analyte binding. Therefore, the ramifications of substrate choice were exacerbated in the case of BCP2 surfaces, where larger *d*_c–c_ were prevalent and a greater proportion of the substrate was exposed.

### 2.2 QCM-D detection of extracellular vesicles using Au NP patterned surfaces

Prior to investigating biosensing with these surfaces, EV-sized particles (ESPs) were first isolated from human plasma using SEC. Fractions were assessed for total protein content and particle concentration (Fig. S5A, ESI†). The highest concentration of ESPs was eluted within fractions 4 and 5. The corresponding protein elution was found to peak later, in fraction 11, confirming that the SEC was effective in separating ESPs from smaller, possibly non-EV artefacts and contaminants. This ensured that the final EV isolate was of sufficient purity to be used for downstream biosensing. The particle size distribution of fraction 4 demonstrates a narrow range. The modal size was determined at 129 nm (Fig. S5B, ESI†), along with the confirmation of EV-enriched proteins CD63 and Alix (Fig. S5C, ESI†).

**Fig. 3:**
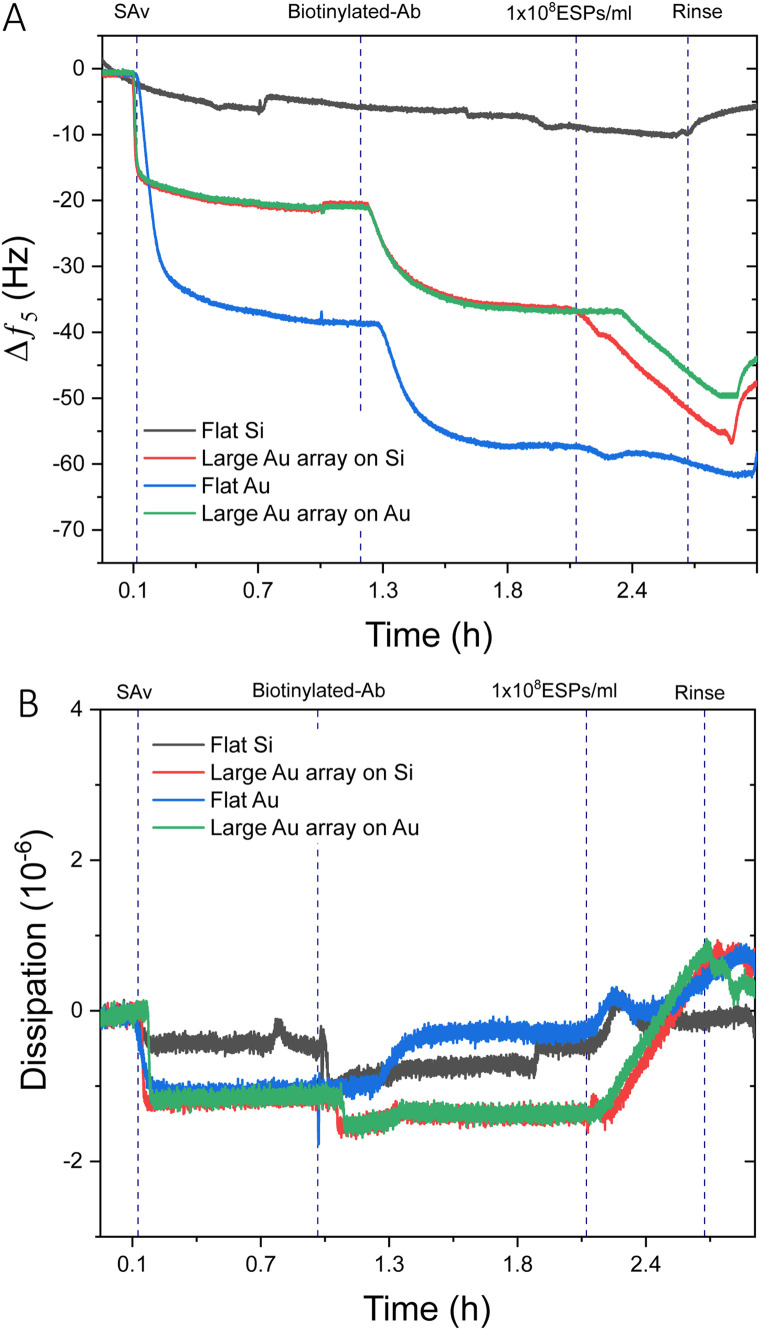
QCM-D analysis of flat sensor surfaces compared to Au NP functionalised surfaces. Example QCM-D (A) frequency and (B) dissipation profiles comparing 1 × 10^8^ ESPs per mL detection on flat gold, flat silica, and large BCP2 Au NP binding arrays on gold and silica.

It was prudent to compare the sensing performance between flat gold and flat silica sensors to silica and gold sensors with large BCP2 Au NPs, to determine whether the introduction of Au NPs influences response to a concentration of ESPs. Fig. 3A displays an example frequency response from the addition of streptavidin (SAv) to ESP detection. As expected, no significant net shifts upon EV addition were seen on the flat silica sensor as the thiol-based affinity fabrication approach was chemically tailored to gold surfaces. Those findings are supported by our characterisation of thiol-based self-assembled monolayer (SAM) formation on silica and gold surfaces, which demonstrated selective chemisorption on gold, with negligible retention on silica surfaces (Fig. S6, ESI†). This is salient when considering where detection may occur on a sensing surface, particularly for gold islands formed on silica. Please note that some irreversible binding of SAv was found on silica surfaces, although lower in magnitude than for surfaces with gold, indicating weak physisorption on the bare silica surface. This may be attributed to differences in the polar component of the surface energies for silica and gold surfaces.^49^

For surfaces with large Au NPs, significantly higher frequency shifts were witnessed upon ESP addition than for flat gold, with Au NPs on gold and silica surfaces exhibiting changes of ∼−5.5 Hz and ∼−9.0 Hz respectively. Dissipation responses were in agreement with the frequency trends (Fig. 3B) with shifts of 1.8 and 2.1 ppm for large Au NP arrays on gold and silica substrates, respectively. These findings are despite a greater mass of SAv and capture antibody binding to the surface of flat gold, as indicated by the larger frequency decrease of ∼−38.0 Hz and ∼−15.0 Hz, respectively. As we will discuss in detail later, various factors may play a role. It is possible that having a denser packing of SAv and antibody molecules may not necessarily result in greater capture of ESPs due to exacerbated steric effects. Steric interactions can occur both between antibodies, preventing their optimal molecular orientation on the sensing surface, as well as between the antibodies and the EVs. Furthermore, binding of particles, such as EVs, with sizes up to 150 nm is known to be limited by inter-particles steric effects.^50–52^

**Fig. 4:**
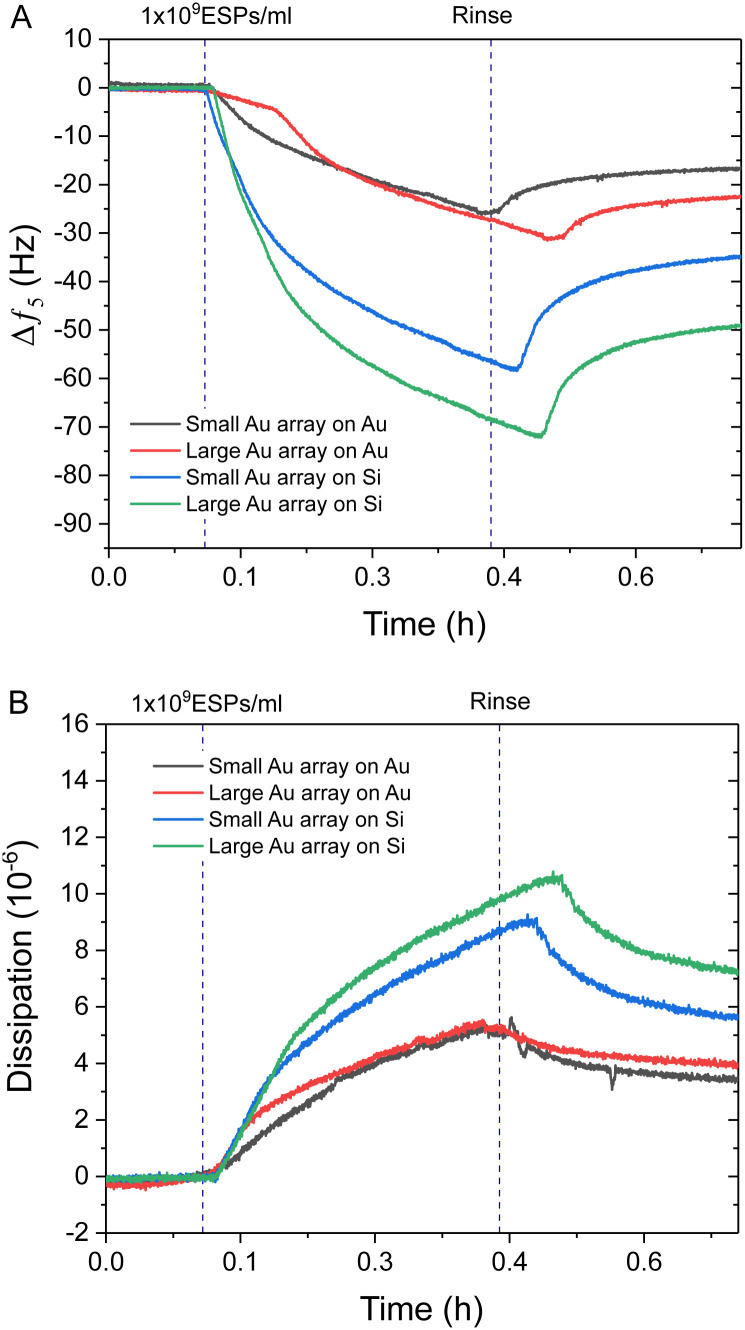
QCM-D analysis comparing to Au NP functionalised surfaces. Example QCM-D (A) frequency and (B) dissipation profiles comparing 1 × 10^9^ ESPs per mL detection with small BCP1 and large BCP2 Au NPs on gold and silica sensors.

Interestingly, Au NPs of both sizes displayed superior binding responses towards ESPs when formed on silica sensors compared to gold surfaces. These results are in spite of the increased theoretical binding surface being offered by the Au NP on gold sensors (Fig. S4C, ESI†). Hereafter, we will define as the theoretical binding surface all the area of the biosensor that is functionalised with gold. However, see the discussion in Section 2.4 for more details, not all of this surface supports binding of the EVs in the same way. Using this definition, an increased binding surface stems from the immunosensor fabrication being specific towards gold surfaces, thus coating with capturing antibodies both Au NPs and underlying Au substrate for gold sensors and only the Au NPs on silica sensors. Therefore, Au NPs on silica offer specific binding regions interspersed amongst non-binding silica domains. This is an important aspect when comparing detection performance between small BCP1 Au NPs and large BCP2 Au NPs, on both gold and silica surfaces (Fig. 4). BCP2 templated gold arrays produced higher changes in frequency towards 1 × 10^9^ ESPs per mL, with ∼−23.0 Hz and ∼−49.0 Hz on gold and silica sensors respectively. Dissipation responses followed a similar trend, with shifts of 4.2 and 7.2 ppm on gold and silica sensors respectively.

The superior performance of Au NPs on silica compared to gold substrates was significant, with even BCP1 Au NPs on silica (∼−35.0 Hz) showing more response than BCP2 Au NPs on gold. Therefore, the addition of non-binding silica spacer regions appeared to be the dominating influence on improving spatial orientation and reduced steric hindrance on ESP binding, with this effect being further enhanced by increasing the Au NP size on silica (Fig. S4C, ESI†). This was in contrast with BCP1-templated arrays, which offered improved binding surface area but denser packing of binding sites. A more detailed analysis of the mechanism leading to enhanced binding will be provided later in the discussion, Section 2.4.

Response curves for the different sensing surfaces were acquired by titrating ESP concentrations, in order to determine changes in sensitivity for frequency and dissipation (Fig. S6, ESI†). The limit of detection (LOD) of each sensor surface in 25% v/v serum is displayed in Table 1. Sensors made of BCP2 Au NPs presented improved LOD compared to BCP1 Au NPs for both sensor substrates. Moreover, Au NPs on silica surfaces demonstrated superior detection of ESPs than the equivalent array on a gold surface. Crucially, despite the larger BCP2 Au NPs on silica possessing 4-fold lower binding surface area (63 400 nm^2^ across a 250 000 nm^2^ reference sensor region) compared to flat gold, the surface exhibited 4-fold higher EV sensitivity for frequency measurements and 1.5-fold higher dissipation performance, indicating superior binding efficiency of the surface that was reflected in a stronger response to all ESP concentrations. The reported LOD was an order of magnitude improvement to a colorimetric sensing approach described by Xia *et al.*^53^ In addition, an advantage of nearly 2 and 3 orders of magnitude was observed compared to interferometric and SPR based detection platforms from Daaboul *et al.* and Liu and colleagues, respectively.^54,55^

**Table 1:** QCM-D sensor surface sensitivity and binding surface area comparison over a reference area of 250 000 nm^2^

Sensor substrate	Gold			Silica	
Structure type	Flat	BCP1 Au NP	BCP2 Au NP	BCP1 Au NP	BCP2 Au NP
Frequency LOD (ESPs per mL)	2.15 ± 0.11 × 10^8^	1.01 ± 0.06 × 10^8^	8.30 ± 0.47 × 10^7^	9.40 ± 0.41 × 10^7^	5.20 ± 0.53 × 10^7^
Dissipation LOD (ESPs per mL)	1.25 ± 0.05 × 10^8^	1.05 ± 0.05 × 10^8^	9.70 ± 0.81 × 10^7^	1.03 ± 0.04 × 10^8^	8.31 ± 0.74 × 10^7^
Binding surface area (nm^2^)	250 000	321 640	273 200	173 130	63 440

Conversely, small Au NP arrays on gold substrates, which provided the greatest binding surface area, did not result in a sensitivity improvement of a similar magnitude, suggesting a comparatively lower detection efficiency of the sensor surface. Interestingly, frequency became the more sensitive mode of measurement upon the introduction of Au NPs, which was contrary to the earlier findings reported.^7^ Linkage or compliance between Au NP and spherical ESP may be more robust than on flat surfaces, with reduced nanoscale rotational and translational motion around the contact region, which could collectively contribute to the reduced dissipation sensitivity.^56^

**Fig. 5:**
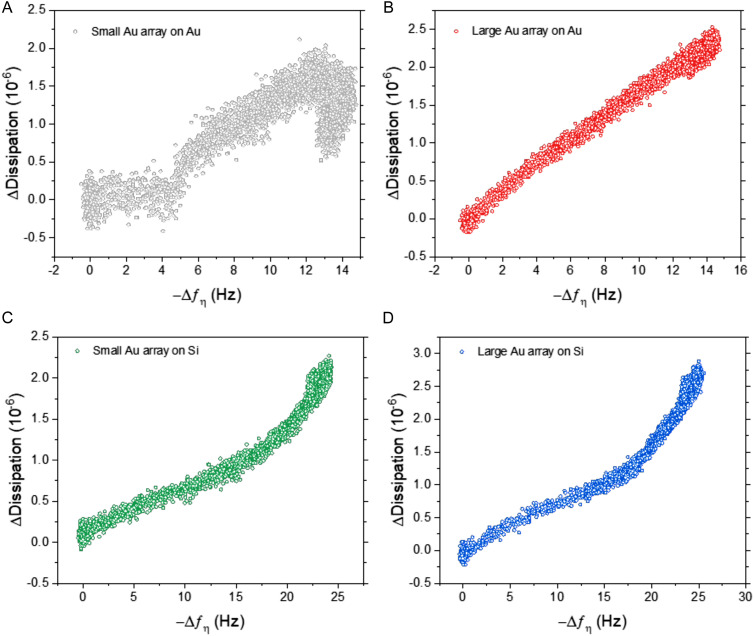
Dissipation change as a function of frequency change towards 1 × 10^9^ ESPs per mL. Response analysis using (A) small BCP1 Au NPs, and (B) large BCP2 Au NPs on silica surfaces, (C) small BCP1 Au NPs, and (D) large BCP2 Au NPs on gold surfaces.

Importantly, the difference in frequency response between silica and gold surfaces was exacerbated at higher ESP concentrations, particularly for the BCP2 Au NP array. This superior dynamic range was further highlighted when comparing the dissipation response as a function of frequency (Fig. 5). All four sensor surfaces were tested with the highest concentration of ESPs (1 × 10^9^ ESPs per mL). Fig. 5A shows that with small Au NPs on Au, there is a lag in response prior to an increase, followed by a reduction in dissipation with no corresponding decrease in frequency. This would suggest that the sensor had become saturated and was undergoing reorganisation of ESPs at the surface to an arrangement that reduced dissipation upon oscillation as the particles enter a more favourable orientation. This aligns with the behaviour witnessed on flat gold surfaces and supports the hypothesis that the density of small Au NPs on Au caused the sensor to perform similarly to flat sensors.^7^ This rearrangement process was also seen with the large Au NPs on Au albeit to a much lesser extent (Fig. 5B). Fig. 5C and D show the opposite effect with silica substrates, where the rate of dissipation response actually increased simultaneously with high frequency shifts, underlining this surface's higher binding capacity at the peak concentrations.

### 2.3 Impact of Au NP array formation on EQCM-D detection of extracellular vesicles

**Fig. 6:**
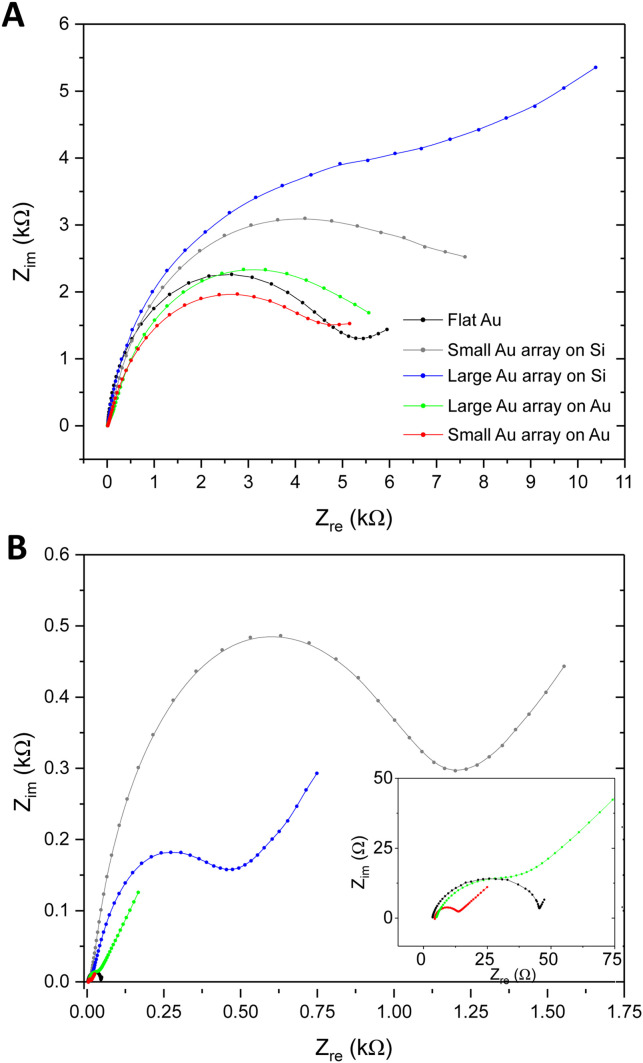
EIS characterisation by Au NP patterned sensors. (A) Nyquist plots in response to 1 × 10^9^ ESPs per mL on Au NP sensors compared to flat gold. (B) Nyquist plots representing inherent EIS of bare sensing surfaces.

Exploration of whether the introduction of nanoarchitectures at the sensor surface could affect the EQCM-D analytical approach was also explored, with a focus on the EIS measurement. Fig. S7A and B (ESI†) illustrate the instrumental setup and the modelling approach employed to execute the EQCM-D characterisation and analyse the impedance data respectively. Fig. 6A presents Nyquist plots as EIS response towards 1 × 10^9^ ESPs per mL using the different Au NP surfaces, which were compared to flat gold. BCP2 Au NPs on silica were found to provide the largest change in impedance upon ESP binding (10.9 kΩ), followed by BCP1 Au NPs on silica (8.2 kΩ). BCP1 Au NP arrays on gold were found to present the lowest changes in EIS and thus the collective results align with the QCM-D responses reported above, with similar theories on ligand orientation and reduced ESP steric hindrance being directly relevant. However, it is important to note that the BCP1 Au NPs on gold displayed lower impedance responses than even flat gold surfaces. To understand this behaviour and to appropriately evaluate total EIS performance, impedance measurements of the bare surfaces were performed, *i.e.* a determination of the native *R*_ct_ of each sensor, without any detection layers or analyte (Fig. 6B).

Sensors with a silica substrate were shown to have a far higher inherent impedance compared to those with a gold substrate. With silica being a poor electrical conductor, the transfer of electrons is confined to the Au NPs on these surfaces, thus increasing the overall *R*_ct_. As we see in Fig. S4C (ESI†), the silica sensor with BCP2 Au NPs displayed a reduced surface area of conductive gold and greater area of silica compared to BCP1 Au NP surfaces, which exhibited a higher gold surface area for electron transfer. For Au NPs on gold, this behaviour was exaggerated, due to the additional availability of conductive gold regions between Au NPs. Clearly, a sensor with a higher gold surface area will provide greater access to electron transfer and thus exhibit far lower EIS *R*_ct_. This is reflected in the results, with the increase in conductive surface area of small Au NPs on gold giving a lower impedance value than both large Au NPs on gold and flat gold itself (inset of Fig. 6B). Therefore, it is possible that the inherent impedance of the surfaces contributed to the EIS changes upon ESP binding in Fig. 6A. Nonetheless, the overall observations confirm BCP2 Au NPs on silica sensors as being the most sensitive platform for EQCM-D detections of CD63-positive extracellular vesicles.

### 2.4 Discussion

Summarising our results, we made three key observations: (A) a higher degree of binding on Au NP surfaces over flat surfaces, (B) a higher degree of binding for Au NPs on silica rather than on gold, and (C) a higher degree of binding on larger Au NPs.

A fully quantitative understanding of the microscopic picture underlying our results necessarily requires detailed molecular modelling, which is outside the scope of this work. Here, we discuss the main physical interactions expected to dominate in this system and show how these can be used to explain, at least on a qualitative level, our observations. In practice, binding of the EVs to the sensor surface requires accounting for (i) the attractive interaction generated by antibody-antigen (CD3) binding and (ii) repulsive steric interactions. The latter include three terms: the steric repulsion between antibodies, the steric repulsion between antibodies and the surface of the EVs, and the steric repulsion between antibodies and the surface of the sensor.

Based on this premise, one could envision two mechanisms consistent with our observations. On the one side, it is well-known that steric hindrance between neighbouring antibodies can lead to a negative cooperativity effect that decreases the effective binding affinity with their receptors.^57^

In practice, this reduction is due to the fact that antibodies must adopt a relatively well-defined orientation to bind their antigen, but this orientation might be hindered by too close neighbours. For this mechanism to occur, the average grafting distance between antibodies (*d*_g_) should be smaller than their lateral size (*L*). For a rough estimation, we take the distance between binding sites on the Y-shaped structure of a typical IgG (antibody) from the Protein Data Bank (PDB) database as *L* = 14.5 nm.^58^ Using our functionalisation protocol, the polyethylene glycol (PEG, 2 kDa) and oligo ethylene glycol (OEG, 800 Da) linkers used to graft streptavidin and spacer ligands have a gyration radius (*R*) that, based on molecular dynamics simulations,^59^ is estimated to be around *R*_OEG_ = 0.9 nm and *R*_PEG_ 1.58 nm, respectively. Because we use a 1 : 9 mixture of streptavidin *vs.* spacer, the average area (*A*) available for each streptavidin, to which the antibody is grafted, is *A*_g_ ≡ 9[(2*R*_OEG_)^2^ + (2*R*_PEG_)^2^] ≈ 41 nm^2^, giving an average grating distance of 
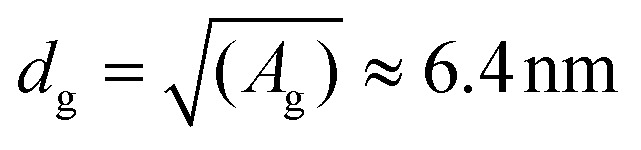
. Because *d*_g_ < *L*, we see that indeed steric clashes between antibodies can occur in our system, backing up the potential for negative cooperativity. Along the same line of reasoning, we also notice that, at equal grafting density, the free volume around a grafted molecule is larger on a curved surface than on a flat one, thereby reducing the effect of steric hindrance. The latter can qualitatively explain the reason why Au NP surfaces have higher binding efficiency than flat ones. In principle, smaller NPs have a higher curvature and thus weaker steric hindrance, hence one could expect enhanced binding. While we expect this to be generally true for Au NPs that are well-spaced, in our case small AuNP are more tightly packed compared to larger ones. For smaller AuNP, the gap between nanoparticles is only 2.6 nm, much smaller than the average size of an antibody. Thus, in this case there are additional steric interactions, both between the antibodies at the periphery of two adjacent nanoparticles and between these antibodies and the adjacent nanoparticles themselves, which further prevent antibodies re-orientation. This steric hindrance is strongly reduced (on gold substrate) or not even present (on silica) for larger NPs, because their distance (18 nm) is much larger than the size of an antibody.

The second potential mechanism that could explain our observation does not depend on interactions between antibodies, but rather on the steric repulsion between antibodies and the EV. This repulsion can lead to a phenomenon, called range-selective binding, whereby two surfaces coated with ligands and receptors can experience a non-monotonic attraction as a function of the number of interacting ligands, *i.e.*, antibodies in our case.^60^ In practice, antibodies that are not bound to CD3 receptors are still acting as a steric barrier protecting the surface of the biosensor from getting in contact with the EV. Increasing the number of antibodies much beyond the total number of CD3 receptors available for binding linearly increases the steric repulsion between the surface of our sensor and the EVs, with a much weaker logarithmic increase in the bond-mediated attraction. Because the total interaction is the sum of these two contributions, and they have opposite character (repulsive *vs.* attractive), an excessive presence of ligands can lead to an overall lower binding strength.^60^ We can again make a back-of-the-envelope calculation using experimental data to estimate the number of antibodies that can potentially interact with an EV (*N*), using formulas from ref. 60. For antibodies grafted on a spherical cap, this number is *N*_abs_ ≈ 2π*RL*/*d*_g_^2^, *R* being the radius of the spherical cap of the nanoparticle, where for simplicity we assumed the EV to be a flat surface, an approximation that becomes more and more accurate the larger the size of the EV compared to that of the AuNP cap. Using the relevant values from our system, we obtain *N*_abs_ = 15 and 65 for small and large nanoparticles, respectively. For a flat surface, whose curvature radius is formally *R* → ∞, we are in the limit *R* ≫ *R*_EV_, where now *R*_EV_ is the radius of an EV, and the correct formula is instead^60^*N*_abs_ ≈ π[*R*_EV_^2^ − (*R*_EV_ − *L*)^2^]/*d*_g_^2^, giving a value of ≈73 for EVs with an average radius of 40 nm like in our system. Based on the experimental data in ref. 61, and considering that only receptors on the lower half of the EV facing the surface can bind to antibodies, we can expect 3 to 4 CD3 interacting receptors per EV. In both cases, this number is much smaller than the total number of interacting antibodies, and we are, thus, in the regime where larger values of antibodies lead to a decrease in binding efficiency. In this regard, it should be noted that although a higher curvature (smaller *R*) reduces the number of antibodies per nanoparticle interacting with the EV, more nanoparticles will be able to interact at the same time with a single EV, due to their smaller size and higher packing. To account for this fact, we estimate the number of interacting antibodies per unit area of the sensor, *σ*_ab_ = *N*_ab_/*D*^2^, with *D* being the centre-to-centre distance between nanoparticles. Inserting the values for our systems, we obtain *σ*_ab_ ≈ 0.03 nm^−2^ and *σ*_ab_ ≈ 0.01 nm^−2^, for smaller and larger nanoparticles, respectively. Thus, we expect a higher binding efficiency for the latter. We also notice that, generally speaking, substituting gold with silica further lowers the number of interacting antibodies per EV, thus reducing steric repulsion and increasing efficiency for the same reasons we have just addressed, an effect that also correlates with our observation.

Finally, it should be noted that a third potential mechanism might not even consider these effects at all, and only rely on the different packing of the EVs adsorbed. On flat and densely functionalised surfaces, EVs can adsorb everywhere and in any random orientation, whereas on periodically structured surfaces there will be preferential adsorption sites and orientations. In 2D, random packing can lead to surfaces with lower packing fractions compared to what can be achieved by more ordered configurations. Although the difference is small, of a few percent only for mono-disperse systems,^62,63^ the effect of anisotropy and polydispersity can increase these differences, especially in the case where adsorption is irreversible.^64^

In practice, in our system it is difficult to tell apart the potential effect of negative cooperativity (which reduces the binding strength of the single antibody-antigen pair) from that of range-selectivity (which reduces the effective interaction mediated by many antibody-antigen pairs, without changing the binding strength of the single pair). In fact, it is arguable that in our system both mechanisms are active, especially considering that they are not mutually exclusive. Furthermore, we note that packing-based effects will only be significant at high surface coverage of EVs, and might not be relevant for the analysis of typical biological samples when the scarcity of EVs prevents this regime from occurring. Instead, negative cooperativity and range selectivity only depend on the grafting density of antibodies, and CD3 receptors, not on the concentration of EVs in the sample. Eventually, all of the aforementioned mechanisms can be active at the same time, and their relative importance in determining adsorption in different regimes can only be assessed by molecular simulations, which constitute an interesting direction for future work.

## 3 Conclusions

This work reports a block copolymer self-assembly fabrication approach to form nanostructured gold arrays from high molecular weight polystyrene-*block*-poly(4-vinylpyridine) thin films with hexagonal architectures, eliciting demonstrable benefit to QCM-D and EQCM-D detection sensitivity and binding efficiency, thereby advancing phenotypic analysis of EVs at concentrations below the clinical range. The introduction of interspersed gold islands increased the platform sensitivity compared to flat gold. Gold arrays matched to the lengthscale of the EVs (diameter *d* = 59.9 ± 8.2 nm; centre-to-centre distance *D*_c–c_ ≈ 74.3 nm) elicited stronger responses than smaller arrays (diameter *d* = 18.4 ± 3.2 nm; centre-to-centre distance *D*_c–c_ ≈ 27.1 nm), with the performance benefits further enhanced when the islands were formed on silica substrates rather than on gold, delivering a limit of detection of 5.2 × 10^7^ ESPs per mL. This represented a 4-fold improvement in sensitivity despite a 4-fold lower surface area available for bioconjugation of antibodies. Similar detection patterns were witnessed for tandem EIS measurements, where the inherent impedance of the underlying silica surface was a key contributor to enhanced EIS sensitivity overall.

Our results suggest a critical role for the spatial organisation of binding sites in optimising EV detection. We relate these findings to their link towards different repulsive (steric) interactions, whose minimisation is necessary to enhance binding efficiency. Grafting of antibodies on discrete and well-separated gold islands on silica reduces steric hindrance between antibodies, and thus negative binding cooperativity, while also lowering the overall steric interaction between antibodies and EVs. Finally, matching of the spatial dimensions of gold islands and EV targets promotes surface adsorption with minimal spatial overlap between EVs, enabling enhanced EV uptake and thus, an extension of the dynamic range.

Our findings provide promising insights into future research directions. Additional work should look to exploit the potential of dual-marker analysis on a single EQCM-D sensing platform through targeted functionalisation of different ligands to silica and gold regions, respectively. Furthermore, we envision a tri-modal analytical approach, by exploiting the newly acquired optical features of the nanoparticle metasurface *via* surface-enhanced Raman Spectroscopy and LSPR,^65^ in addition to the acoustic and electrochemical analysis. In combination with the potential integration of EV isolation *via* electrokinetic trapping (enabled by a nanoisland architecture),^66,67^ the approach presented herein provides promising pathways into holistic solutions for use in disease diagnostics.

## 4 Experimental section

### 4.1 Materials

For surface characterisation, Si (100) wafers (p-type) were purchased from MicroChemicals GmbH and cut to appropriated dimensions (1 × 1 cm). Two different poly(styrene)-*block*-poly(4-vinylpyridine) (PS-*b*-P4VP) materials with *M*_w_ = 22 kg mol^−1^ (*M*_w_ PS = 15 kg mol^−1^; *M*_w_ P4VP = 7 kg mol^−1^) and *M*_w_ = 257 kg mol^−1^ (*M*_w_ PS = 187 kg mol^−1^; *M*_w_ P4VP = 70 kg mol^−1^) were purchased from Polymer Source, Inc., Canada, and were used without further purification. Propylene glycol methyl ether acetate (PGMEA) (reagent plus, 99.5%), THF (ACS reagent, 99.9%), and tetrachloroauric acid (HAuCl_4_) (99.999% trace metals basis) were purchased from Merck and used without further purification. For isolation and sample preparation, qEV original SEC columns (Izon Science, UK), human plasma (Sigma Aldrich, USA), 0.45 μm filters (Merck Millipore, USA), HEPES buffered saline (HBS, 0.01 M HEPES, pH 7.4, 0.15 M NaCl) (GE Healthcare Life Sciences, Sweden) and Amicon Ultra-15 centrifugal filters (Merck Millipore, USA) were used. For sample characterisation 100 nm polystyrene beads (Thermofisher Scientific, UK), a microBCA assay (ThermoFisher, UK) and RIPA buffer (Sigma Aldrich, USA) were acquired. For QCM detection, mouse monoclonal biotinylated-anti CD63 (353017, Biolegend UK), biotin-IgG isotype control antibody (400103, Biolegend UK) and streptavidin (Sigma Aldrich, USA) were utilised. For EQCM-D measurements, materials included; ferrocyanide (98.5%, Honeywell), ferricyanide (99%, ACROS Organics), gold-coated and silica-coated QCM sensors from QuartzPro, Sweden.

### 4.2 EV isolation and characterisation

#### Size-exclusion chromatography

SEC was implemented as the EV isolation technique from human plasma. The source plasma was first filtered with a 0.45 μm filter (Merck Millipore, USA). 30 mL of clarified plasma was subsequently concentrated using Amicon Ultra-15 centrifugal filters with a 10 kDa pore size cut-off (Merck Millipore, USA). The filters were spun at 4000*g* for 30 minutes at 4 °C. Post-spin, 0.5 mL of concentrated filtrate was loaded onto a qEV 35 nm SEC column (Izon Science, UK). 0.2 μm filtered HEPES buffered saline (HBS, 0.01 M HEPES, pH 7.4, 0.15 M NaCl) (GE Healthcare Life Sciences, Sweden) was used as the eluting buffer at a flow rate of 1 mL min^−1^. 20 × 1 mL fractions were collected and stored at −80 °C.^7^

#### Protein analysis of isolates

Total protein concentration of SEC isolates was determined using the micro-BCA protein assay as per manufacturers instruction (see ESI,† for details). Validation of the SEC protocol was conducted by verifying EV presence through western blot analysis using capillary gel electrophoresis. Based upon previously reported SEC isolation protocols, SEC fraction 4, was considered for onward protein characterisation for EV presence.^68,69^ EV proteins Alix (97 kDa) and tetraspanin CD63 (57 kDa) were probed by chemiluminescent immunoassay, using mouse monoclonal anti-Alix and mouse monoclonal anti-CD63 as primary antibodies. The WES run was conducted as per the manufacturer's instruction (see ESI,† for details).

#### NTA analysis of SEC fractions

Based upon the protein identification from the western blot, the fourth isolation fraction was chosen for concentration and hydrodynamic size characterisation of particulates nanoparticle tracking analysis (NTA) with the Nanosight LM10 instrument (Malvern Instruments, UK). The machine was calibrated with 100 nm polystyrene beads (Thermofisher Scientific, UK) prior to fraction assessment. Measurement specifications were as follows: 532 nm green laser, 5 videos per fraction, 60 second video length, shutter speed of 25–32 ms, camera gain of 400, camera level 15, lower threshold of 910 and higher threshold of 11 180. Captured videos were processed using the NTA software version 3.2, a detection threshold of 5, auto settings for blur, minimum track length and minimum particle size. Measurements were carried out in static mode at room temperature.^7^

### 4.3 PS-*b*-P2VP BCP self-assembly

Hexagonal out-of-plane cylindrical arrays formed from BCP self-assembly in thin films were produced by spin coating a 2 wt% solution of PS_15K_-*b*-P4VP_7K_ (BCP1) in PGMEA and a 2 wt% solution of PS_187K_-*b*-P4VP_70K_ (BCP2) in a mixture of PGMEA : THF (90 : 10 volume ratio) respectively onto bare silica and gold coated QCM sensors. Coating was performed at 3000 rpm, for 30 s following cleaning with oxygen reactive ion etching (RIE) plasma for 90 s.

The choice of solvent for the polymer solution is dictated by the material-solvent parameters (affinity and evaporation rate) and the macromolecular characteristics of the respective BCP system. Both THF and PGMEA are suitable solvents for PS and P4VP blocks respectively according to their Hansen solubility parameters.^70^ Despite THF presenting a higher affinity for both blocks than PGMEA, the latter appeared to be a better option for the adopted BCP system since it presents a higher boiling point than THF (146 °C *vs.* 66 °C). This could support slower solvent evaporation during the spin-coating process, giving more mobility to the polymer chains and supporting self-assembly. The increase in the *M*_w_ for BCP2 (PS_187K_-*b*-P4VP_70K_), can reduce this polymer chain mobility, solubility and inhibit fast microphase separation.^71,72^ Thus the addition of a small fraction of THF likely supported solvation for the casting solution.

### 4.4 Selective Au impregnation and Au array formation

1 wt% solutions of HAuCl_4_ in Milli-Q water were used to impregnate the BCP films by overnight immersion of the sample in the solution. The silica and gold surfaces were exposed to oxygen reactive ion etching (RIE) plasma treatment in a Diener Electronic PICO instrument (Germany) (plasma conditions: 20 sccm O_2_, 60 s) in order to etch the polymer scaffold and reduce the gold salts to Au^0^.

### 4.5 Surface characterisation

#### 4.5.1 Atomic force microscopy

AFM images of surfaces after spin coating and after plasma etching were obtained using a Bruker Dimension Icon instrument (UK) with a Bruker ScanAsyst Air probe (normal tip radius 2 nm, spring constant = 0.4 N m^−1^, resonance frequency = 70 Hz in air) in ScanAsyst mode at 1 kHz oscillation and a linear scan rate of 0.5 Hz. Images were processed using WSxM software (version 5.0)^73,74^ Height and diameter profiles of particles were taken as an mean from 15 detected particles across 3 micrographs. Centre-to-centre distribution and fast Fourier transform (FFT) was determined using WSxM PSD and FFT options respectively. Particle diameter was determined using Pebbles software.^75^

#### 4.5.2 Grazing-incidence small-angle scattering

GISAXS experiments were performed using a SAXSLab Ganesha 300XL (8 keV), as part of the CNIE research facility service, University College London. The incident angle was set at 0.18°. 2D scattering patterns were collected with a PILATUS 300 K solid-state photon-counting detector at a sample-to-detector distance of 1400 mm. GISAXS data analysis was performed using FitGISAXS software.^76^ Au NPs formed on Si surfaces after etching BCP1 and BCP2 were used for analysis to exploit scattering differences between Au NPs and the Si substrate, since it would be challenging to detect Au NPs on Au substrates.

#### 4.5.3 Spectroscopic ellipsometry

An optical study of the Au NP decorated silicon-wafers and plain silicon-wafers was performed using spectroscopic ellipsometry (Semilab SE2000, Hungary)) at an incident angle of 73°. Obtained values for *Ψ* and Δ were subsequently analysed using the Semilabs SEA software (v1.6.2).

### 4.6 EQCM-D

All QCM-D measurements were performed on a Q-Sense E4 instrument (Biolin Scientific, Sweden). Analysis of frequency and dissipation response was conducted using the QTools software, version 3.0.17.560 (Biolin Scientific, Sweden). Changes in resonance frequency (Δ*f*) were recorded from the third, fifth, seventh, ninth and eleventh overtones. The presented data relates to the 5th overtone, with variation of (Δ*f*) between overtones being 10% or less. In all instances, samples were degassed prior to exchange in the QCM flow module and AT-cut, 5-MHz gold or silica coated quartz crystal sensors with a 0.79 cm^2^ active area (Biolin, Sweden) were used.

All EIS measurements reported herein were conducted using a Q-Sense Electrochemistry Module from Biolin Scientific (Sweden), in tandem with a Q-Sense Analyser instrument and a Gamry (UK) Ref. 600 Plus potentiostat. The system used QCM sensors as the working electrode (WE), a platinum counter electrode (CE) and Ag/AgCl reference electrode (RE) as part of a conventional three-electrode system. Data was acquired using Gamry Instrument Framework (v7.07) software and analysed using Gamry Echem Analyst (v7.07) software.

EIS experiments were all carried out with a frequency scan range of 10^−1^ Hz to 10^5^ Hz at a 5 mV AC amplitude. The detection area was set at 0.79 cm^2^. An equimolar solution of 5 mM of K_3_ [Fe(CN)_6_]/K_4_ [Fe(CN)_6_] in 0.1 M KCl was used for all measurements. Modified Randles cell circuit models used to fit against EIS data were explored and an optimal model was selected. Impedance was determined after the formation of each layer, following the addition of electrolyte into the chamber. The flow of electrolyte through the chamber was paused during measurement acquisition. EIS were captured in tandem with QCM-D response in all instances.^8^

To ensure reproducibility of each process, all analytes were prepared using the same degassed stock solutions to minimise impact of buffer properties during sample exchange in observed responses. These were prepared to identical volumes (0.25 mL per sensor). All reagents were sourced from the same suppliers throughout the study to avoid influences of differing characteristics or quality. In all cases, the analyte was flowed at 10 μl min^−1^ and a sensor was reserved for baseline measurement, to account for drift and background changes induced by buffer exchange. Frequency and dissipation responses are reported net or post-HBS rinse, to account for the removal of weakly bound analytes.^7^

#### 4.6.1 QCM-D immunosensing of CD63-positive extracellular vesicles

An affinity-based immunosensing approach was employed as reported by Suthar *et al.*^7^ A 1 mM ethanolic solution of SH-PEG (2 kDa)-Biotin and spacer molecule SH-OEG (800 Da)-COOH at a 1 : 9 mol mol^−1^ ratio was flowed across the sensor surface at 7.5 L min^−1^ overnight to form a self-assembled monolayer (SAM). Subsequently, a 100 μg per mL solution of streptavidin (SAv) was flowed across the sensor surface at 10 μL min^−1^, followed by a rinse step of HBS buffer at 80 μL min^−1^. 20 μg per mL of mouse monoclonal biotinylated anti-CD63 was immobilised on the surface at 10 μL min^−1^, followed by another rinse step and response stabilisation for 30 minutes prior to sample addition. Thus only gold regions across the surface were modified for sensing, whilst any underlying silica substrate remained unfunctionalised. The sensing capabilities of flat (unmodified) silica and gold-coated surfaces were subsequently compared to silica and gold surfaces which had been modified with large Au NPs following the impregnation of BCP2-based templates. The sensors were assessed against 1 × 10^8^ ESPs per mL in 25% v/v serum.

Sensitivity of silica and gold coated sensors, modified with small and large Au NPs following impregnation of BCP1- and BCP-2 based templates were then compared. The sensors were assessed against the following concentrations: 5 × 10^7^, 7.5 × 10^7^, 1 × 10^8^, 2.5 × 10^8^, 5 × 10^8^, 7.5 × 10^8^ and 1 × 10^9^ ESPs per mL in 25% v/v serum. LOD and LOQ of the sensor surfaces were determined by replacing the anti-CD63 antibody with a biotin-IgG isotype control antibody. LOD and LOQ were defined as the minimum ESP concentration displaying an SNR of 3 and 10 respectively.^77^ SNR was determined as a ratio of the response elicited on the target and control sensor surfaces using the concentrations provided above.

#### 4.6.2 EQCM-D analysis of CD63-positive extracellular vesicles

The presence of Au NPs on both silica and gold sensors, allows for detection of CD63-positive extracellular vesicles using electrochemical impedance as a comparison technique to QCM-D. Impedance was measured for all four sensor surfaces against 1 × 10^9^ ESPs per mL in 25% v/v serum and compared against impedance values from a flat (unmodified) gold sensor. In order to determine the influence of the sensor surface on the overall impedance value, the measurement protocol was conducted on the bare sensor surfaces post-Au NP formation, without the addition of any of the immunosensing layers. The inherent impedance of the four sensor surfaces was again compared to that of a flat (unmodified) gold sensor.

## Conflicts of interest

There are no conflicts to declare.

## Supplementary Material

NH-008-D2NH00424K-s001
