# Supplementary material for: Amplified EQCM-D detection of extracellular vesicles using 2D gold nanostructured arrays fabricated by block copolymer self-assembly
Source: Nanoscale Horiz. 2023 Jan 24;8(4):460–72. doi: 10.1039/d2nh00424k (PMC10042438; doi:10.1039/d2nh00424k)
Supplement: NH-008-D2NH00424K-s001 [file NH-008-D2NH00424K-s001.pdf]

Electronic Supplementary Material (ESI) for Nanoscale Horizons.
This journal is © The Royal Society of Chemistry 2023

—– SUPPLEMENTARY MATERIAL—–

Amplified EQCM-D detection of extracellular vesicles using 2D gold nanostructure arrays fabricated by block copolymer self-assembly

Jugal Suthar,[a,b] Alberto Alvarez-Fernandez,[*a] Esther Osarfo-Mensah,[a] Stefano Angioletti-Uberti,[b] Gareth R. Williams,[c] Stefan Guldin[*a]

Total protein assay

Total protein concentration of SEC isolated EVs was determined using the micro-BCA protein assay (Thermo-Fisher Scientific, UK). Briefly, 5 µl of EV sample was incubated for 15 minutes on ice with 15 µl of RIPA buffer. The sample was then sonicated for 5 minutes, before 10 µl of the lysed solution was pipetted into a 96-well plate. The micro-BCA working reagant was created and incubated with the sample as per manufacturer's instructions. The final absorbance readings were measured at 562 nm.

Western Blot methodology

40 µl of deionized water was added to dithiothreitol (DTT) to make a 400mM solution. 10 µl of SEC fraction 4 was lysed in 20 µl RIPA buffer as described above. The lysed solution was then diluted to a working concentration between 0.2-1.0 µg per µl with 0.1X sample buffer, before 4 µl was mixed with 1 µl of loading buffer (flourescent 5x master mix). The loading buffer was prepared by adding 20 µl of 10x sample buffer, and 20 µl of 400 mM DTT solution. Since the detection of tetra-spannin proteins requires non-reducing/native conditions, an additional 20 µl of 0.1X sample buffer was added in place of the DTT solution for these samples. Thus, Alix and CD63 were detected in separate capillaries. The samples and biotinylated ladder were then denatured on a 95 °C heat block for 5 minutes, before being briefly centrifuged and loaded onto the detection module assay plate. Anti-alix and anti-CD63 primary antibodies were used at 1:20 and 1:10 dilutions respectively and loaded onto the primary antibody. Corresponding mouse-reactive secondary antibodies were then added, followed by the chemi-luminescent substrate, comprising 200 µl luminol-s and 200 µl peroxide. The fully loaded plate was then centrifuged at 2000RPM for 5 minutes before being inserted into the WES system, in conjunction with a 13-capillary cartridge. Detection and quantification was conducted via a CCD camera and the Compass software, version 3.1.7 (Protein Simple, USA). Anti-Calnexin (W17077C, Biolegend) was used as a negative control to ensure that no cellular protein was present in the chosen SEC fraction.

[a] Department of Chemical Engineering, University College London, Torrington Place, London, WC1E 7JE, U.K. [b] Department of Chemical Engineering, University College London, Torrington Place, London, WC1E 7JE, United Kingdom. [c] UCL School of Pharmacy, University College London, 29-39 Brunswick Square, Bloomsbury, London, WC1N 1AX, United Kingdom. *e-mail: alberto.fernandez@ucl.ac.uk; s-guldin@ucl.ac.uk.

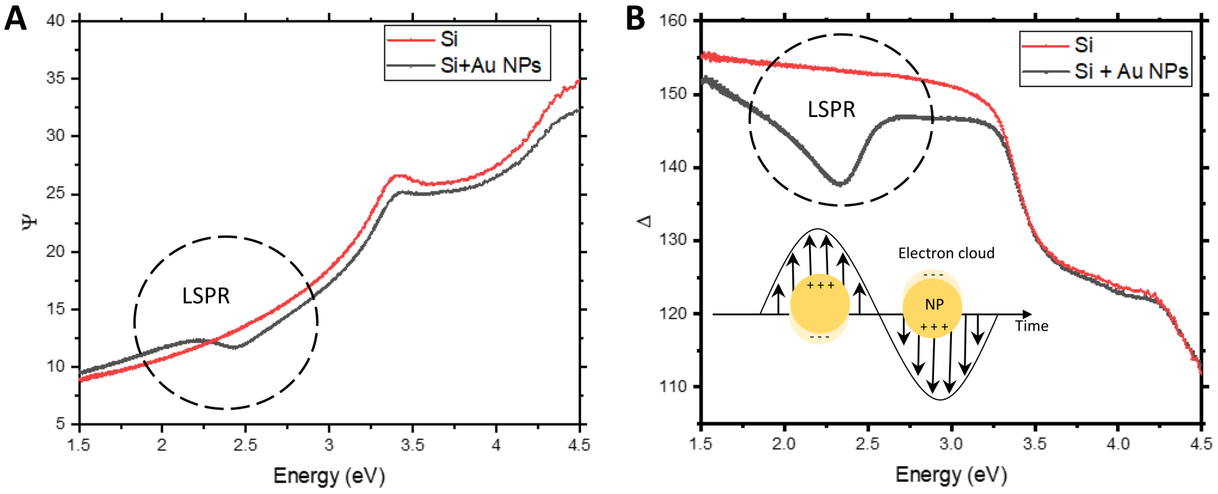

Supplementary Figure S1 | Post-RIE spectroscopic ellipsometry results obtained from a bare and BCP2 Au NPs functionalised silicon wafers. (A) Ψ and **(B)** Δ as a function of photon energy. Observed plasmon resonance is circled on both.

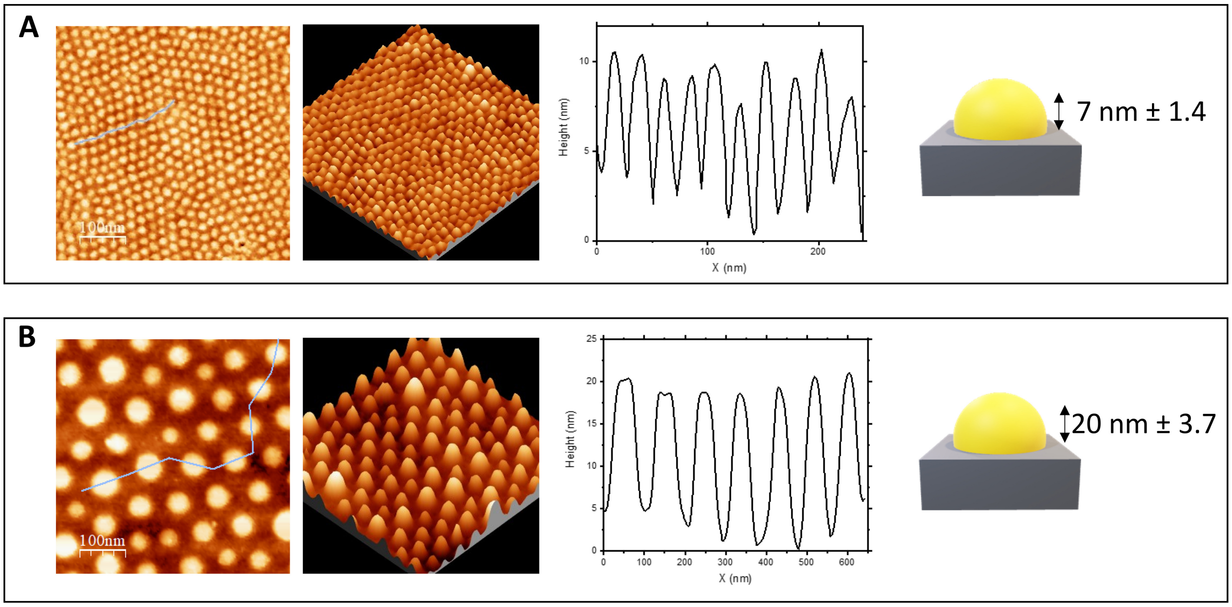

Supplementary Figure S2 | Out-of-plane AFM micrographs of Au NPs silica sensors post-RIE etch. AFM topographical profile analysis of Au NPs via 2D and 45° tilted 3D AFM micrographs, including schematic of Au NP height for surfaces formed from **(B)** BCP1 and **(B)** BCP2 templates. Scale bars: 100 nm.

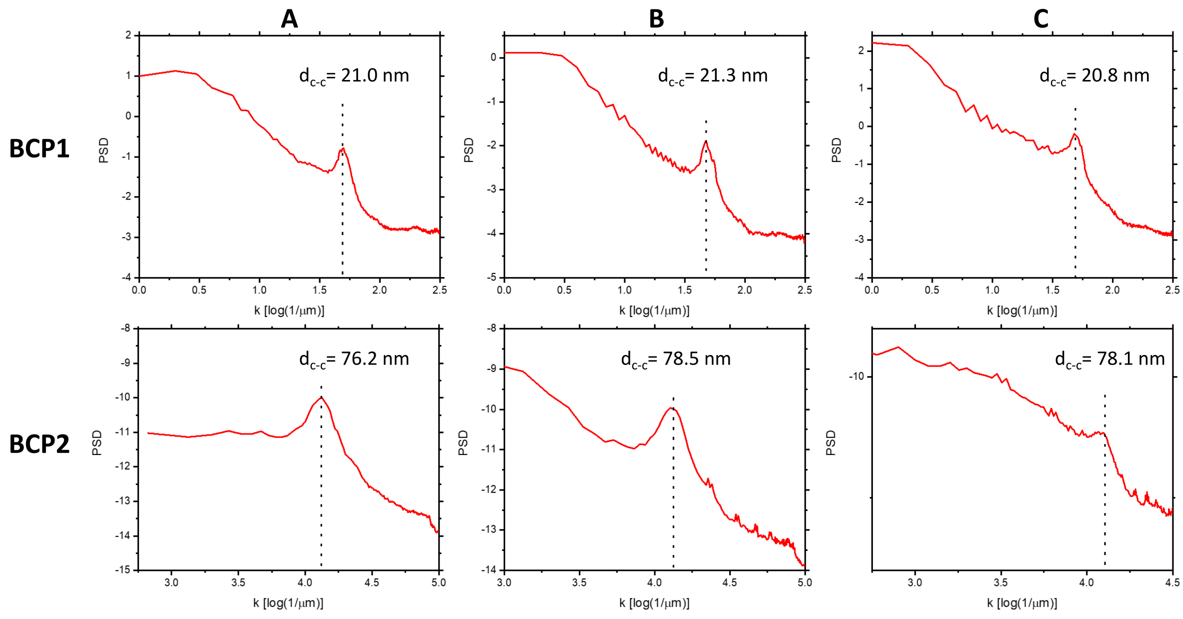

Supplementary Figure S3 | Particle size distribution (PSD) analysis of sensor surfaces during Au NP fabrication process. PSD graphs calculated as the square of the absolute value of the FFT, determining estimated centre-to-centre diseases of features formed from BCP1 and BCP2 templates following **(A)** BCP self-assembly, **(B)** gold impregnation into P4VP domains following overnight incubation and **(C)** removal of PVP template with oxygen plasma etching.

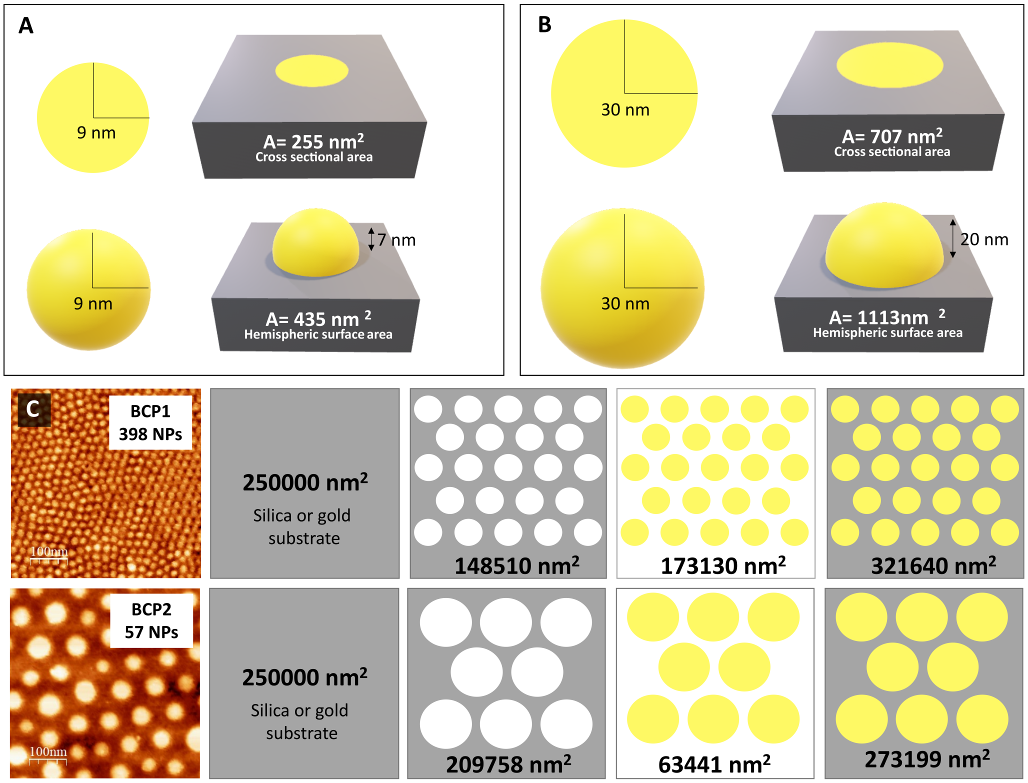

Supplementary Figure S4 | Estimation of Au NP size, density and surface area. AFM-derived surface area estimation of **(A)** BCP1 and **(B)** BCP2 Au NPs. **(C)** Schematic representation of sensor surface area across 500 nm^2 region, with and without Au NPs, and Au NPs alone, derived from both BCP1 and BCP2 templates. Scale bar: 100 nm.

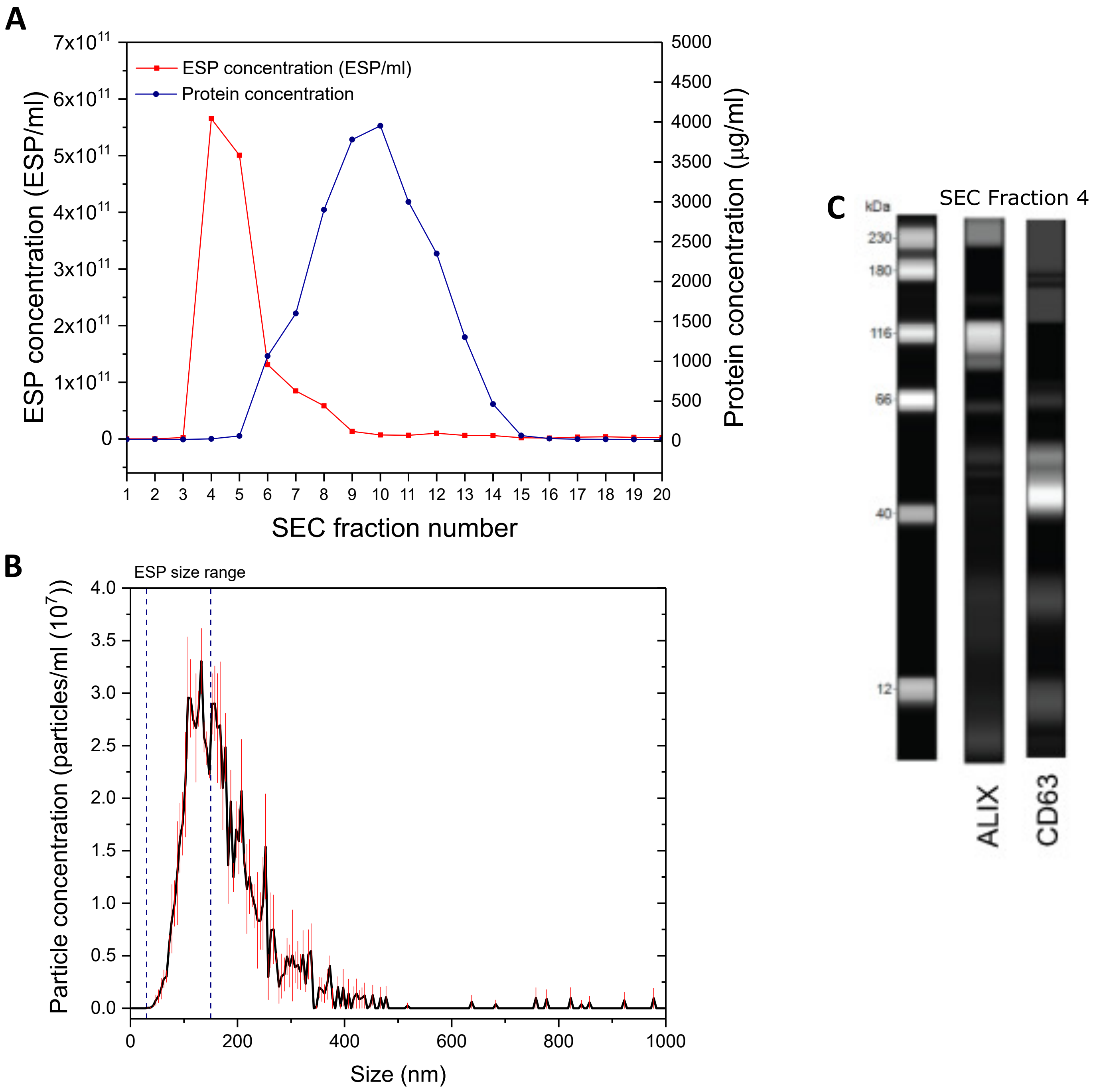

Supplementary Figure S5 | Particle size characterisation of ESP SEC isolation. (A) ESP and protein concentration profiles across SEC fractions using NTA and microBCA analysis. **(B)** Particle size distribution of SEC fraction 4. **(C)** Western blot identification of CD63 and Alix proteins in SEC fraction 4.

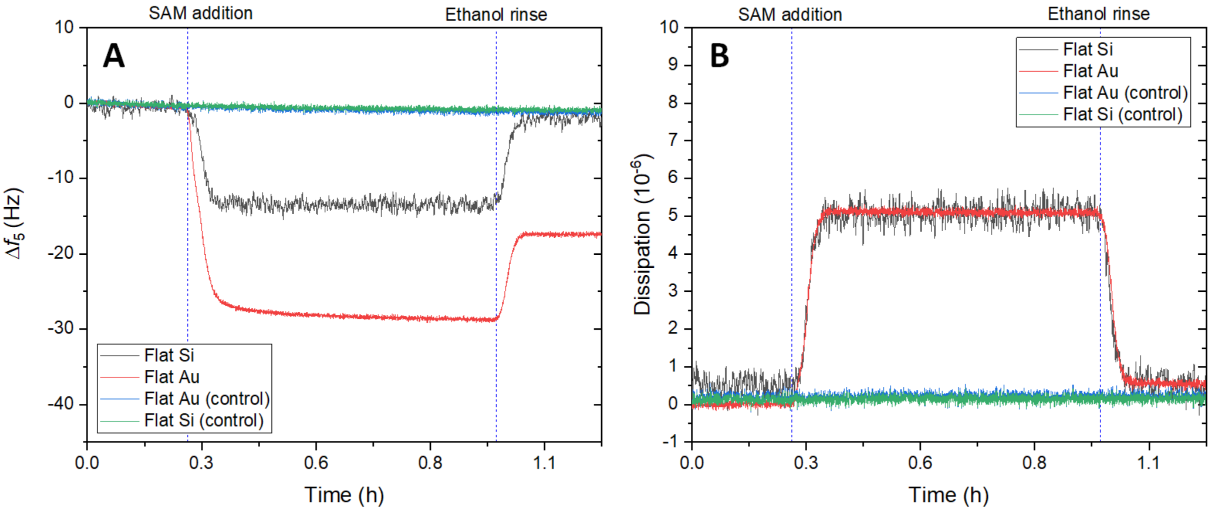

Supplementary Figure S6 | SAM formation characterisation on gold versus silica sensors using QCM-D (A) frequency and **(B)** dissipation plots for the SAM formation of 1 mM ethanolic solution of SH-PEG(2 kDa)-Biotin and SH-PEG-COOH in a 1:9 ratio, with flow rates of 77.5 µl/min. The Flat Si and Flat Au traces represent exposure to a PEG thiol containing solution, whilst the Flat Au (control) and Flat Si (control) had a only 95% ethanol solution with no PEG thiol. This discerned any environmental influences on SAM formation. The addition of the ethanolic SAM solution led to a negative change in the frequency, and an increase in dissipation. This demonstrates adsorption of the dissipative PEG thiols to the sensors surfaces. Rinsing the sensors with 95% ethanol led to desorption of unbound material from surfaces. The silica coated sensor returned to baseline values, demonstrating that the changes seen in the frequency and dissipation were the result of weaker physisorption. Compared to the baseline, the frequency values for the gold sensor showed the retention of mass at the surface, coupled with the slightly elevated dissipation indicating the presence of chemically bound PEG chains via the thiol groups.

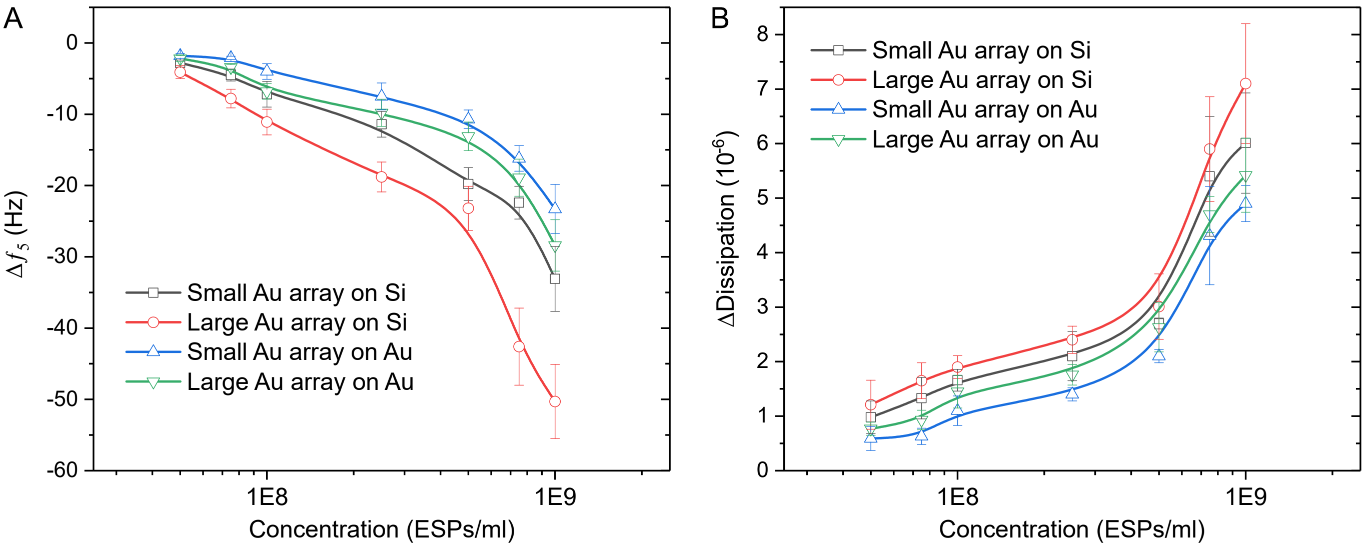

Supplementary Figure S7 | QCM-D concentration response curves to CD63-positive extracellular vesicles on Au NP functionalised sensors. QCM-D **(A)** frequency and **(B)** dissipation response curves, comparing detection of varying concentrations of ESPs with large BCP2 and small BCP1 Au NPs on gold and silica sensors. Standard deviation determined from three independent experiments.
